# An unusual case of persistent groin pain after total hip arthroplasty: a case report

**DOI:** 10.1186/1752-1947-5-67

**Published:** 2011-02-15

**Authors:** Praveen Konala, Thomas K Schaefer, Farhad Iranpour, Niklaus F Friederich, Michael T Hirschmann

**Affiliations:** 1 Musculoskeletal Surgery Department, Imperial College, London, UK; 2 Department of Orthopaedic Surgery and Traumatology, Kantonsspital Bruderholz, CH-4101 Bruderholz, Switzerland

## Abstract

**Introduction:**

Arthroplasty is a well-established routine elective surgical procedure in orthopaedics. To a great extent, diagnosis, treatment and post-operative rehabilitation in these patients is standardised. In a busy clinic, surgeons from time to time tend to focus their attention on common causes of joint pain, but it may lead them to overlook sinister but less common pathologies. Here we report a case of a patient with groin pain due to pre-operatively undetected pelvic metastases from a pyeloureteral carcinoma who underwent total hip arthroplasty. There are several case reports which deal with primary or secondary tumours which were either discovered at the time of replacement surgery or developed at the site of prosthesis years after total hip or knee replacement. To the best of our knowledge, this is the first case report in which a metastatic cancer was missed pre-operatively and intra-operatively both by the radiologist and by the orthopaedic surgeon and should be reported so that surgeons are reminded to be careful when dealing with seemingly routine cases.

**Case presentation:**

A 79-year-old Caucasian woman presented to the arthroplasty clinic with groin pain. Initial radiographs showed subtle bilateral abnormalities in the pelvis. Neither the radiologist nor the orthopaedic surgeon recognized it. A diagnosis of osteoarthritis of the hip was established, and she underwent total hip arthroplasty. Despite initial improvement, the patient came back with worsening hip pain three months later. Further radiological examination revealed multiple metastatic lesions throughout the pelvis due to a pyeloureteral carcinoma.

**Conclusions:**

This case report emphasizes the importance of meticulous, unbiased pre-operative assessment of patients and their radiographs, even in so-called routine clinical cases. Often subtle radiological changes are classed as normal, especially if they are bilateral. Further radiological imaging should be recommended in all cases where unexplained clinical features or radiological findings are present.

## Introduction

Total hip arthroplasty (THA) is a well-established routine surgical procedure in orthopaedics [1-3]. To a great extent, diagnostics, treatment and postoperative rehabilitation in these patients are standardised [1-3]. In a busy hip clinic, surgeons from time to time tend to focus their attention on common causes of hip pain, but it may lead them to overlook other sinister but less common pathologies.

Here we report a case of a patient with groin pain due to pre-operatively undetected pelvic metastases from a pyeloureteral carcinoma who underwent THA. There are several case reports that deal with primary or secondary tumors which have either been discovered at the time of replacement surgery or developed at the site of prosthesis years after total hip or knee replacement [4-9]. To the best of our knowledge, this is the first case report in which a metastatic cancer was missed pre-operatively and intra-operatively both by the radiologist and by the orthopaedic surgeon and should be reported so that surgeons are reminded to be careful when dealing with seemingly routine cases.

Upper urinary tract tumours are uncommon and constitute 5% to 7% of all urinary tract tumours [10]. A ratio of 56% to 98% of patients with pyeloureteral carcinoma present with microscopic or macroscopic haematuria [11]. Around 30% present with flank pain, and 19% present with features of advanced disease, including bone pain, anorexia and weight loss. The thin layer of smooth muscle around the upper urinary tract predisposes these patients to early local invasion and metastasis. Urothelial tumours spread by local invasion as well as lymphatic and haematogenous spread. The incidence of bone metastasis in upper urothelial tumours is hard to compute in view of its rarity and the lack of large data sets. Studies of metastatic urinary tract tumours by Sengeløv *et al*. [12,13] showed that the most common sites of metastasis are lymph nodes (26%-57%) and bone (35%-40%). Among those with bone metastasis, the spine was involved in 40% of the cases, followed by pelvis in 26% [12,13].

## Case presentation

**Figure 1:**
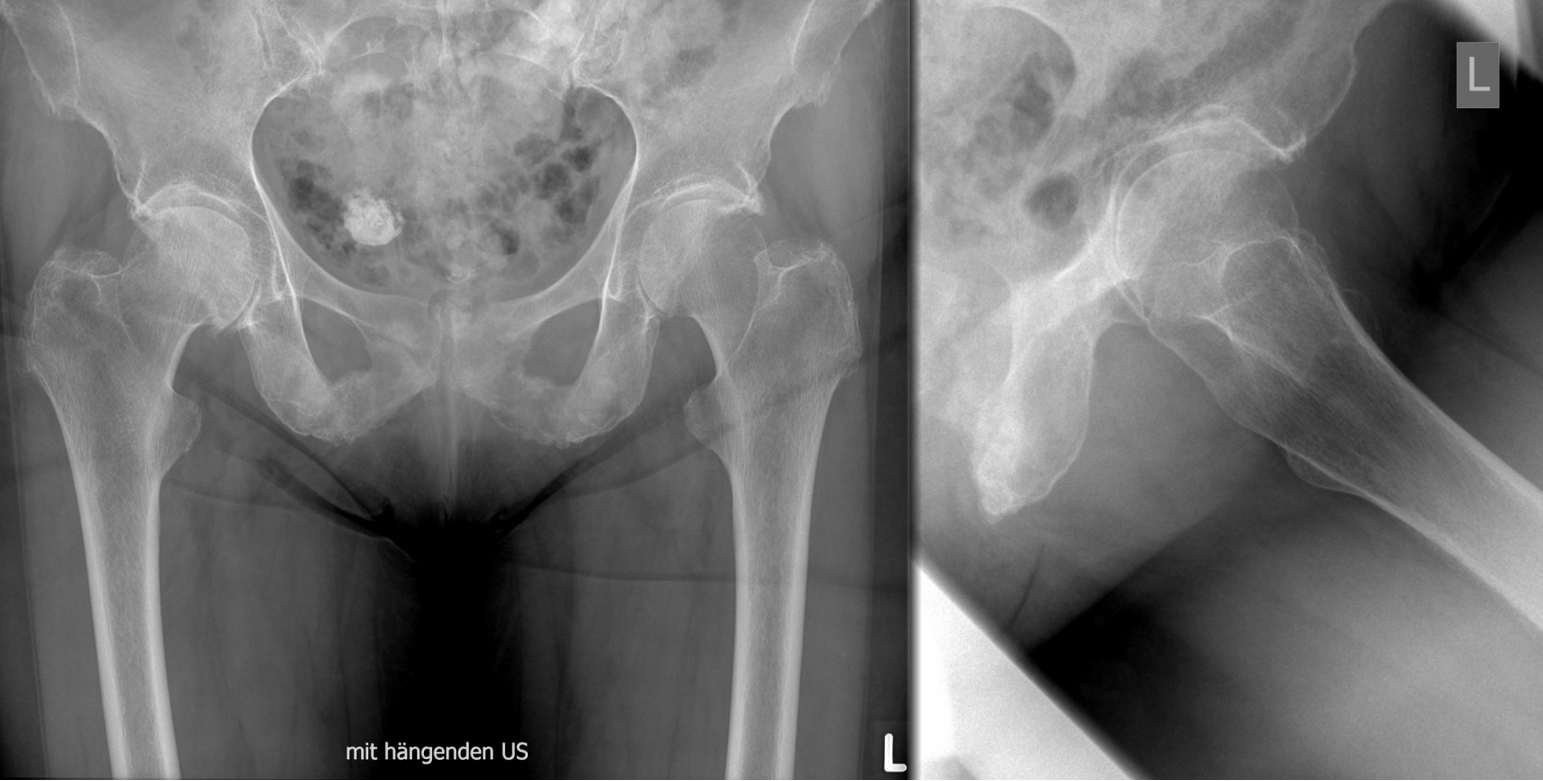
**Pre-operative antero-posterior pelvic (left) and true lateral (right) radiographs of the left hip showing bilateral osteoarthritis of the hip and a calcified uterine myoma**.

**Figure 2:**
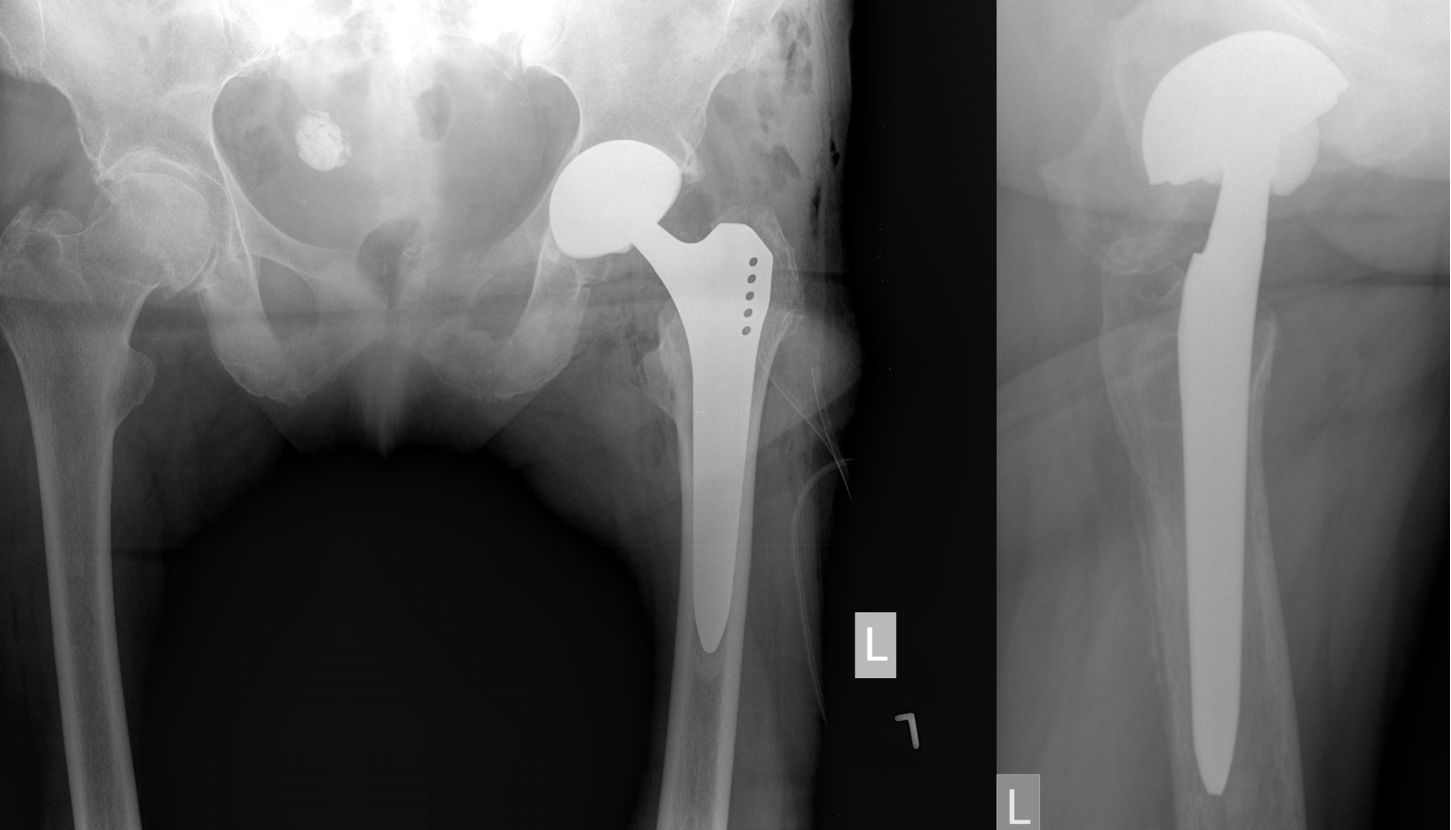
**Initial postoperative antero-posterior pelvic (left) and true lateral (right) hip radiographs with acceptable implant position**.

**Figure 3:**
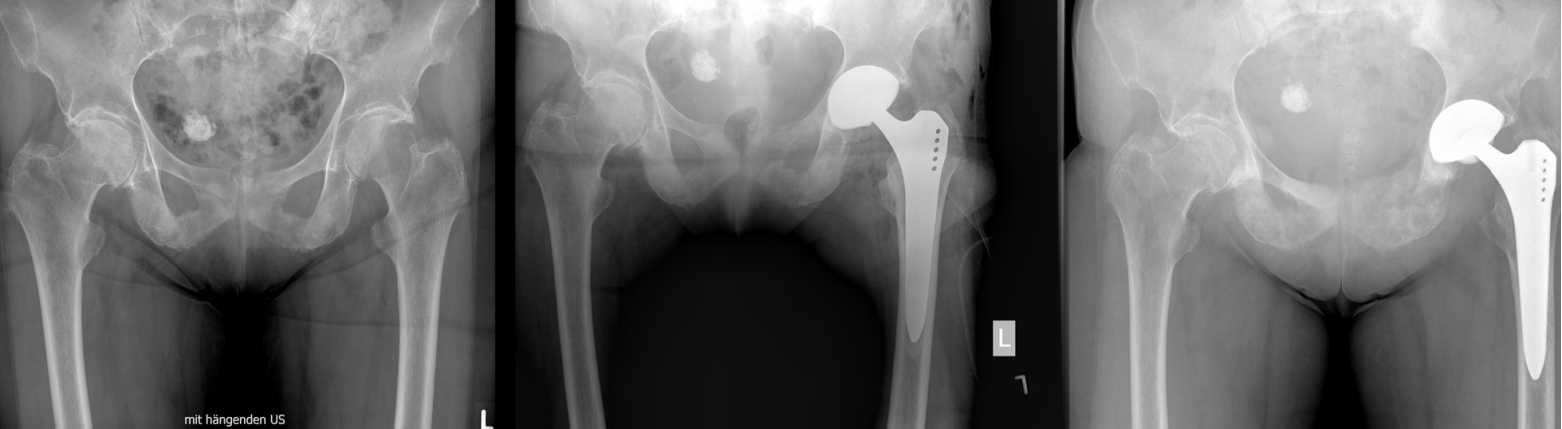
**Comparison of anteroposterior pelvic hip radiographs preoperatively and six weeks and three months post-operatively (from left to right)**.

**Figure 4:**
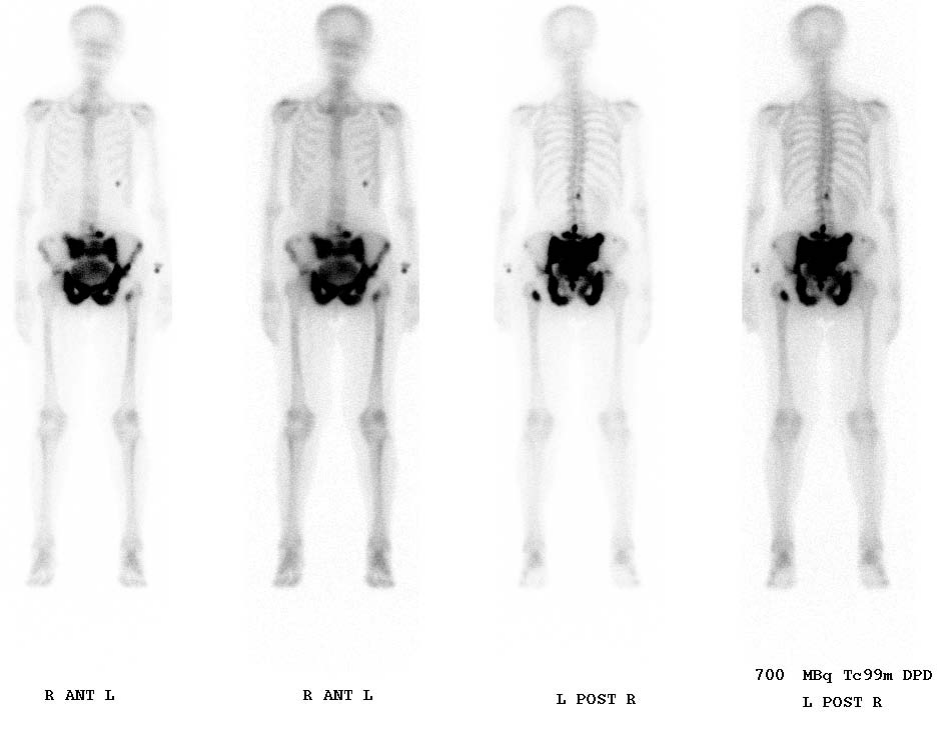
**A technetium-99 m bone scan revealed widespread osteolytic and osteoblastic lesions in the entire pelvis, spine and chest**.

**Figure 5:**
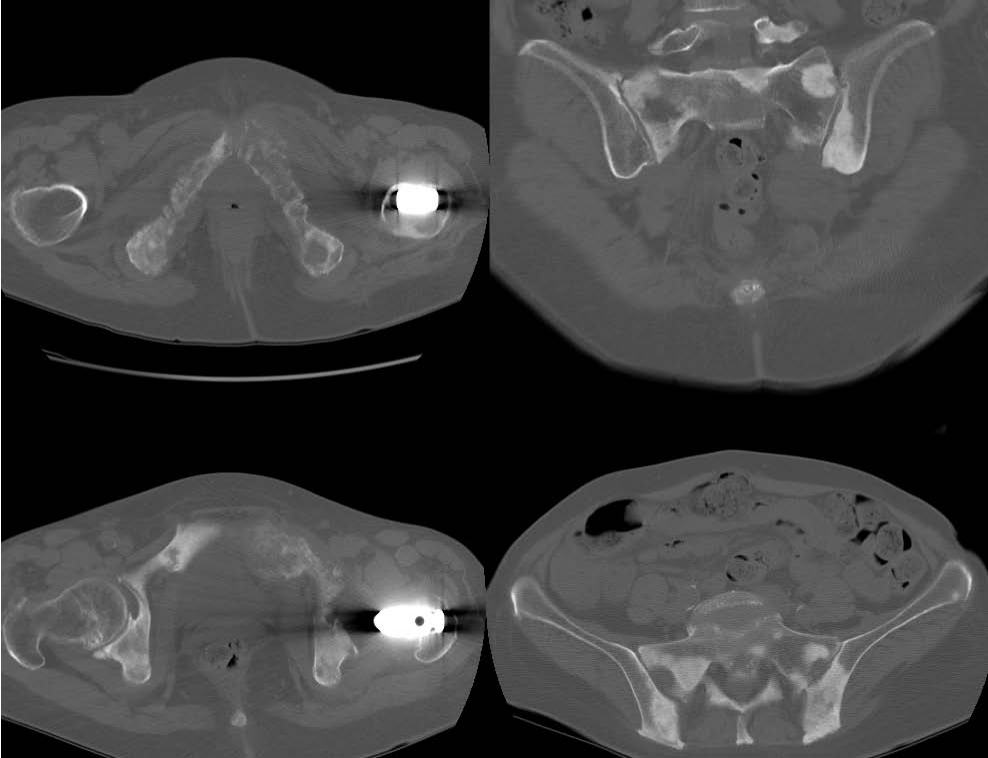
**Computed tomography confirmed the widespread osteolytic and osteoblastic lesions in the entire pelvis, spine and chest**.

A 79-year-old, active Caucasian woman with left groin pain was referred to the orthopaedic clinic by her general practitioner to undergo THA. She complained about progressive left groin pain for 12 months that was worst in the morning and exacerbated by physical activity and prolonged sitting. Clinical examination revealed left-sided antalgic gait and tenderness in her left groin. Left hip internal rotation was limited to 10°. Anteroposterior pelvic and true lateral hip radiographs revealed typical signs of osteoarthritis (Figure 1). In addition, there was a 25-mm-diameter calcification in the lesser pelvis, which was reported by the radiologist to be a calcified uterine myoma. Finally, the patient was scheduled for elective left THA. At the preadmission clinic, a routine urine dipstick test revealed 3 to 20 erythrocytes and no leucocytes or nitrites. On the basis of the urine dipstick stick, a diagnosis of urinary tract infection (UTI) was made, although the patient was asymptomatic and urine was sent for culture. The patient was empirically treated with oral antibiotics, and the surgery was postponed. Urine cultures showed no bacterial growth, and no further tests or referral to other specialties were done. Four weeks later she underwent a THA with satisfactory recovery. At the first follow-up six weeks postoperatively, the patient was almost pain-free and was using two crutches for stability. Her examination was unremarkable at that time. Standard radiographs showed an acceptable implant position (Figure 2). The orthopaedic surgeon and the radiologist did not report any other abnormality. Further physiotherapy was recommended, and routine follow-up was recommended six months from the time of surgery. Unexpectedly, the patient presented to us four months after surgery complaining of worsening left hip pain. Because she was still on crutches, she noted weakness, loss of appetite and weight loss of 5 kg. Further examination revealed generalized tenderness of the left iliac crest, gluteal region and groin. The radiographs then showed bilateral cloudy bone formation in the pelvis (Figure 3). Further investigation with a technetium-99 m bone scan and computed tomography (CT) revealed widespread osteolytic and osteoblastic lesions bilaterally in the superior and inferior pubic ramii, sacrum, iliac wings, acetabula and left transverse process of the L4 and L5 vertebrae, which were most likely metastatic (Figures 4 and 5). Screening for the primary tumor (CT of the chest abdomen and pelvis) revealed a left pyeloureteral carcinoma. No further invasive tests or histopathological examinations were done because of the advanced stage of the disease, and a decision was made to provide palliative treatment to the patient. The patient received palliative chemotherapy and radiotherapy and died one year after diagnosis.

## Discussion

Many case reports have been published with regard to tumours developing at the site of arthroplasty [4-9]. It may be assumed that in these cases, the tumour was already present pre-operatively and could have been detected by more extensive clinical and radiological investigation. In our case, even retrospectively it is hard to pick up the pathology on the initial pelvic radiographs, but a closer look reveals an abnormal texture of both pubic bones. This was considered normal, however, as it appeared bilaterally. In addition, the calcified myoma distracted the surgeons' and radiologists' attention. A comparison with a normal pelvic radiograph from another patient could have been helpful and might possibly have resulted in an earlier diagnosis.

Interestingly, the patient's surgery was postponed because of microscopic haematuria, which was interpreted as a lower UTI. However, haematuria is one of the most common findings in urothelial carcinoma, although it is not specific and is also present in cases of UTI [14]. The question whether our patient's preoperative symptoms were caused in total or at least in part by the osteoarthritis is difficult to answer, but the initial clinical presentation as well as the fact that the patient was temporarily pain-free after THA points in that direction. During surgery, no abnormal bone presentation was detected in the hip joint, which further supports this theory, although it is highly likely that the metastases were present at the time of initial presentation. Whether the surgery may have led to a more rapid progression of the metastatic disease because of compromise of the immune system remains unclear. On this issue, no evidence is available in the literature.

## Conclusion

This case report is of particular importance to all radiologists and any surgeon involved in elective orthopaedic surgery. It highlights the importance of avoiding the pigeon-holing of patients with specific symptoms into specific diagnostic categories to correctly diagnose the outliers. Further, radiographs should be analyzed in a systematic, standardized and complete manner, taking every visible structure into account. In the presence of bilateral subtle abnormalities, further radiological imaging such as magnetic resonance imaging or single photon emission tomography should be recommended. In conclusion, the importance of an unbiased assessment of patients and their radiographs is a *sine qua non *for the establishment of the correct diagnosis.

## Consent

Written, informed consent was obtained from the patient for publication of this case report and accompanying images. A copy of the written consent is available for review by the Editor-in-Chief of this journal.

## Competing interests

The authors declare that they have no competing interests.

## Authors' contributions

PK reviewed the case and drafted the manuscript. FI participated in drafting the manuscript and case review. TS participated in drafting the manuscript and literature review. NFF participated in drafting the manuscript and case review. MTH reviewed the case and drafted the manuscript. All authors read and approved the final manuscript.
